# Competency-based mental health supervision: evidence-based tool needs for the humanitarian context

**DOI:** 10.1017/gmh.2022.23

**Published:** 2022-05-10

**Authors:** Bettina Böhm, Miguel Palma, Janet Ousley, Gregory Keane

**Affiliations:** Médecins Sans Frontières, Paris, France

**Keywords:** Clinical supervision, competency-based supervision, mhGAP, task-sharing, task-shifting

The mental health (MH) care needs in humanitarian crises often far outweigh the local workforce capacity, making task-sharing to non-specialist MH staff essential to provide care for people with severe and common mental disorders (Murray and Jordans, 2016; Singla *et al*., 2017; Patel *et al*., 2018; Carreño *et al*., 2019). Yet, task-sharing strategies cannot (and should not) replace professional accreditation models involving years of training. Clinical supervision of MH staff in task-sharing contexts is widely accepted as a way to guarantee quality of care; however, these activities are not consistently prioritized by MH policy makers who may focus on quick fixes such as one-off trainings without follow-up strategies including supervision, or on the immediate needs of the health care system over the needs of frontline care providers (Kemp *et al*., 2019). In low-resource and humanitarian settings (LRHS), this means that practitioners with limited experience often care for high numbers of extremely vulnerable patients after time-limited training (Faregh *et al*., 2019), with expert support only provided occasionally (Kemp *et al*., 2019). Typical capacity building approaches have focused on ensuring fidelity to intervention manuals. The emphasis must shift, however, to building practical clinical skills over time.

Clinical supervision from a senior MH professional is critical for effective knowledge and skills transfer to novice practitioners (Holt *et al*., 2015; Faregh *et al*., 2019), thereby ensuring that they can provide evidence-based interventions safely. Although not replacing the imperative need to scale up psychosocial support to MH professionals, clinical supervision is also central in helping supervisees manage their own reactions to patients by addressing issues such as emotional over-involvement in or disengagement from a therapeutic process (Brockhouse *et al*., 2011), which can harm patients. Protecting staff psychosocial well-being is particularly important in conflict, post-conflict, epidemic and disaster settings where they themselves are exposed to substantial and often ongoing suffering. Leaving their occupational stress unaddressed may lead to staff loss and high turnover, with consequent drops in care quality, shortages in and interruptions of essential services, which ultimately affects service users.

Successful supervision in LRHS is complicated by the fact that evidence-based practices have largely been developed for professionals in high-resource environments with none of the realities or constraints of humanitarian crises, which can include limited expert clinical care and supervision. Firstly, supervisory senior staff may be the only formally trained clinician for a large population, and any time spent supervising others can take away from their own patient or client services. Secondly, consistency of supervision is a challenge. Regular staff turn-over is exacerbated by supervisors having different theoretical orientations, disparate supervision styles, and sometimes limited experience supervising others. A corollary of these factors, inadequate or poorly conducted supervision therefore results, for example, in decreased empathic engagement in patient care (Rønnestad *et al.*, 2019) or a reduction in regular opportunities for shared reflection and improvement, harm to clinicians and clinical malpractice. It is therefore crucial to ensure structure and consistency to supervision with concrete guidance and practical tools. These should be evidence-based (Holt *et al*., 2015), conducive to learning (Kemp *et al*., 2019), culturally appropriate (Heim and Kohrt, 2019) and context-adapted to different global expressions of suffering and forms of healing (Faregh *et al*., 2019; Heim and Kohrt, 2019; Kohrt *et al*., 2020*a*). Finally, co-design and field testing of these instruments may enable an easier institutionalization of competency-based supervision models.

Médecins sans Frontières (MSF), a non-profit humanitarian organization, prioritizes clinical supervision as an essential component of MH and psychosocial support activities in LRHS, building upon existing competency-based training approaches (that have been applied by MSF to the mhGAP intervention guide, e.g.). However, with respect to competency-based supervision, we have observed a dearth of available resources relevant to the contexts where we have operations. To fill this gap, MSF has designed tools to support professionals delivering supervision. Evidence-based competencies for provision of psychiatric, psychological or counselling care were initially identified (see research manuscript). Prioritization was given to common factors of the therapeutic alliance rather than to the technical components of a particular intervention. Abstract concepts such as hope or empathy, which are common factors of quality therapeutic consultations (Singla *et al*., 2017; Pedersen *et al*., 2020; Kohrt *et al*., 2020*a*) featured in task-sharing manuals, had to be turned into tangible-specific behaviours. These represent standards with the goal of accommodating the multitude of professional, educational and cultural backgrounds found in LRHS. This frame allows supervisors with different theoretical backgrounds to set consistent learning objectives, and in doing so enables supervisees to build on previously developed skills. Additionally, the use of structured case formulation discussions helps to build understanding of vulnerability and resilience (Sørbye *et al*., 2019) to produce more patient-centred care. To our knowledge, no tool for monitoring case formulation within supervision is available for non-specialists.

We hope that MSF's recent effort is a start. Yet, while we realize that one ideal tool for every context may not be feasible, a broader availability of tools may enable supervisors to better address the specificities of their contexts while maintaining a focus on evidence-based treatment and incorporating standardized manuals (e.g. mhGAP). This should also reduce the need for duplication between organizations. We eagerly await WHO's EQUIP package for psychological interventions (Kohrt *et al.*, 2020*b*) and invite critical feedback on the MSF toolkit, which can be found here: https://scienceportal.msf.org/assets/7610. We hope that sharing our experience can help refine current and future supervision approaches, and we invite other global MH actors to design and share their tools to improve quality of care for some of the world's most vulnerable patients.
