# Competency-based mental health supervision: evidence-based tool needs for the humanitarian context – ERRATUM

**DOI:** 10.1017/gmh.2022.31

**Published:** 2022-06-14

**Authors:** Bettina Böhm, Miguel Palma, Janet Ousley, Gregory Keane

The publisher apologises that upon publication of Böhm, B., Palma, M., Ousley, J., & Keane, G. (2022) the link to the tool being discussed was missed off and should have appeared as below:

We eagerly await WHO's EQUIP package for psychological interventions (Kohrt et al., 2020b) and invite critical feedback on the MSF toolkit, which can be found here: https://scienceportal.msf.org/assets/7610

The online version of this article has been updated.
